# SIVA1 Knockdown Drives Aggressive Phenotypes in Triple-Negative Breast Cancer Cells while Enhancing Paclitaxel Efficacy

**DOI:** 10.1021/acsomega.6c03785

**Published:** 2026-06-20

**Authors:** Natália Sudan Parducci, Bruna Oliveira de Almeida, Anali Del Milagro Bernabe Garnique, Maria Fernanda Lopes Carvalho, Isabelle Diccini, Leticia Veras Costa-Lotufo, João Agostinho Machado-Neto

**Affiliations:** Department of Pharmacology, Institute of Biomedical Sciences, 28133 Universidade de São Paulo, São Paulo CEP 05508-900, Brazil

## Abstract

SIVA1 is a multifunctional protein involved in processes that are critical for tumor progression. In breast cancer, *SIVA1* is frequently overexpressed; however, its functional role remains poorly defined. This study aimed to elucidate the cellular mechanisms regulated by SIVA1 and to characterize its molecular network in breast cancer. Integrated *in silico* analyses and *in vitro* functional and molecular assays in breast cancer cell lines were conducted to investigate *SIVA1* expression and associated signaling pathways. Cell proliferation, migration, invasion, and drug sensitivity were assessed in 2D and 3D culture models. Molecular analyses included markers of DNA damage and apoptosis, cytoskeletal regulators, ubiquitination status, and p53 signaling. The subcellular localization of SIVA1 was investigated. *SIVA1* was overexpressed in breast cancer cells and associated with pathways related to protein secretion, spliceosome activity, DNA repair, and oxidative stress. Functional analyses linked *SIVA1* to mitochondrial permeabilization, ubiquitin-mediated processes, cytoskeletal regulation, and p53-dependent apoptosis. *SIVA1* knockdown increased cell growth, migration, and invasion, while enhancing sensitivity to paclitaxel in both 2D and 3D models, evidenced by reduced ATP levels and increased cleaved PARP1 and γ-H2AX. *SIVA1* knockdown resulted in the accumulation of phosphorylated p53 and reduced phospho-Stathmin 1 and ubiquitin levels. SIVA1 displayed predominant nuclear localization, independent of treatment. In summary, these findings identify *SIVA1* as a key regulator of proliferation and cytoskeletal dynamics in breast cancer, whose inhibition induces a more aggressive yet taxane-sensitive phenotype, with potential implications for patient stratification and therapeutic decision-making.

## Graphical abstract

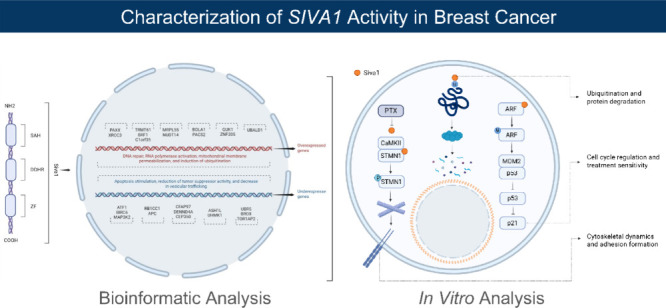

## 1 Introduction

Recent forecasts underscore growing concerns in public health regarding cancer progression, a group of diseases that affected over 19.3 million people and caused 10 million deaths in 2020 alone,^1^ numbers that reflect population aging, lifestyle changes, and expanded diagnostic coverage.^1,2^ Cancer incidence varies widely across types and regions, influenced not only by biological but also socioeconomic and political factors.^3^ Among these, breast cancer stands out due to its high mortality in advanced stages, especially in low- and middle-income countries.^4^ Recognized as the most prevalent cancer among women, its triple-negative subtype is characterized by an unfavorable prognosis, decreased survival rates, and limited responsiveness to current therapies.^5–7^

Therapeutic development benefits from dissecting cellular and molecular pathways that shape disease behavior, which is key to increasing treatment specificity.^5,8^ One hallmark of cancer is the disruption of cellular growth, division, and death, governed by complex gene-protein networks.^9^ Among these genes, *SIVA1* has attracted interest for its role in cell survival across diverse tumors, including osteosarcoma, hepatocellular carcinoma,^10^ lung,^11^ and breast cancers.^12^

SIVA1 mediates its effects via a broad network of signaling molecules. In cell death, it acts through receptors like CD27 and TNFRSF18 and interacts with XIAP, NF-κB, BCL-2, and JNK2,^13–15^ establishing a dual activity: via extrinsic (receptor-mediated) and intrinsic (mitochondrial/caspase) apoptotic pathways.^16–18^ Its strong expression during p53-driven apoptosis and its capacity to trigger cell death highlight its potential role as an apoptotic pathway effector.^19–22^

Accumulating evidence indicates that SIVA1 plays complex and sometimes opposing roles in tumor biology. In several cancer models, SIVA1 has been implicated in key processes such as apoptosis, metastasis, invasion, and drug resistance,^23,24^ although its functional impact appears to be highly context-dependent.^25^ Some studies describe SIVA1 as a pro-apoptotic factor, thereby promoting apoptotic signaling and suppressing metastatic traits, as observed in triple-negative breast cancer (TNBC),^26,27^ in cervical cancer,^13^ and in leukemia models.^28,29^ Conversely, other reports suggest that SIVA1 activity may support tumor progression in certain molecular contexts,^11,30^ highlighting the dual nature of its biological functions.

Despite these observations, the specific role of SIVA1 in breast cancer remains poorly defined. Notably, transcriptomic analyses indicate that *SIVA1* expression is elevated across multiple molecular subtypes of breast cancer compared with normal mammary tissue;^31^ however, its functional contribution, particularly in TNBC, remains insufficiently characterized. This gap highlights the need for a clearer understanding of the biological role of SIVA1 in breast cancer and provides a rationale for the present study.

Our results demonstrate that *SIVA1* is a critical regulator of p53 and stathmin 1 (STMN1) activity in TNBC, with protein levels correlating with major biological processes such as proliferation, migration, invasion, and spheroid formation. Notably, reducing *SIVA1* expression improved paclitaxel (PTX) efficacy and attenuated malignant features, suggesting its potential utility for patient stratification.

## 2 Results and Discussion

### 2.1 *SIVA1* Expression is Elevated in Tumor Samples and Shows Association with DNA Repair, Translation, Ubiquitination, and Apoptosis in the Breast Cancer Cohort

The analysis of *SIVA1* gene expression across 23 cancer types, including both solid and hematological tumors, revealed a widespread upregulation in tumor samples compared to normal tissues with a particularly pronounced increase in the breast cancer cohort (Figure S1a). Considering the potential relationship between *SIVA1* expression and disease development risk, prognostic investigations were conducted in The Cancer Genome Atlas (TCGA) breast cancer cohort. Analyses revealed no statistically significant association between *SIVA1* expression levels and overall (*p* = 0.72; HR = 1.05), disease-specific (*p* = 0.61; HR = 0.89), disease-free (*p* = 0.39; HR = 0.81), or progression-free survival (*p* = 0.45; HR = 0.88), as illustrated in Figure 1a.

**Figure 1:**
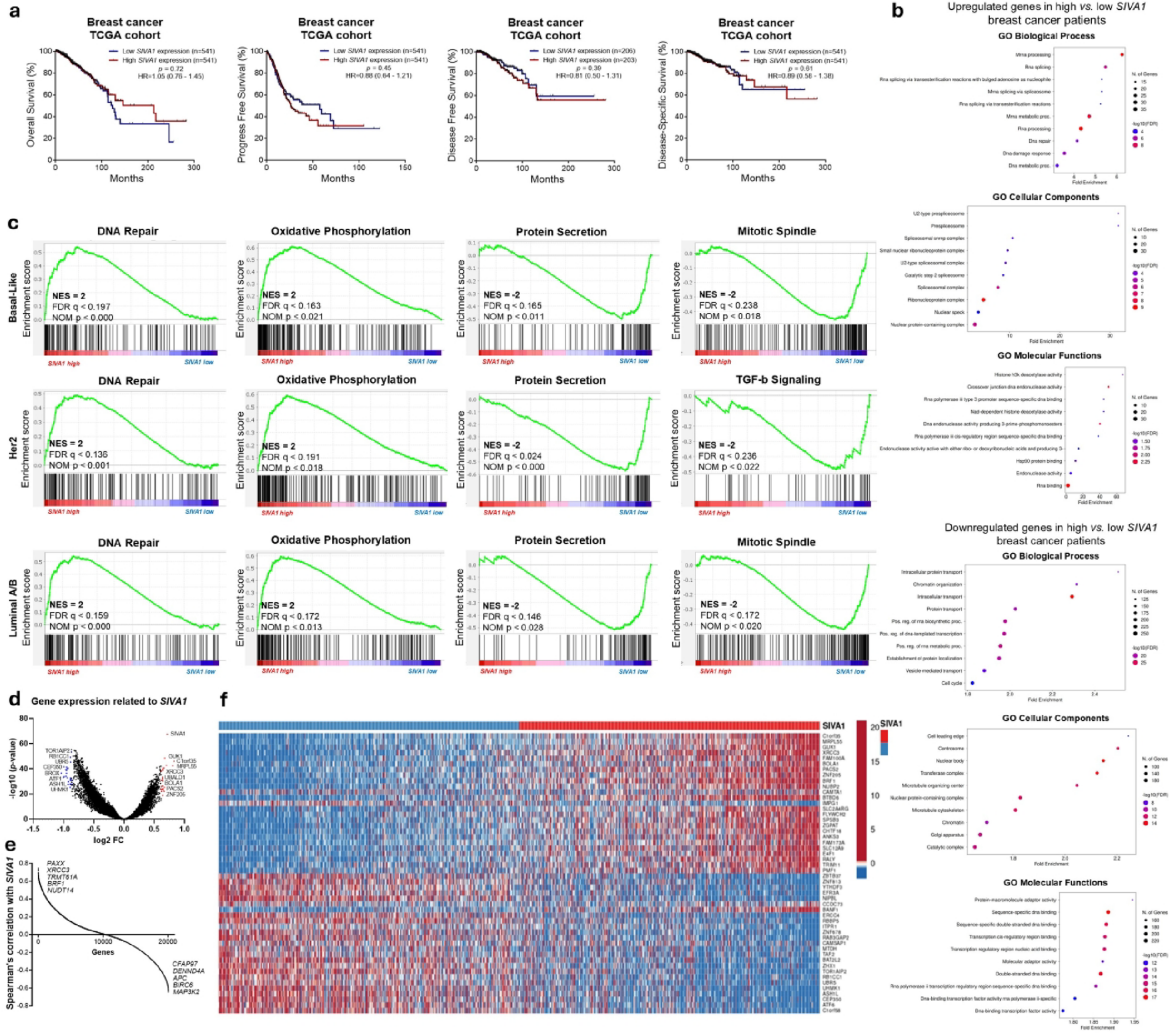
*SIVA1* expression is increased in breast cancer tumor samples and interferes with mitochondrial, cytoskeletal, and DNA repair system activities. (a) Clinical information and *SIVA1* expression data in breast cancer patients were obtained from the cBioPortal platform. The Invasive Breast Carcinoma TCGA PanCancer Atlas cohort (*n* = 1082) was selected and dichotomized in high (*n* = 541) and low (*n* = 541) gene expression based on the median. Associations between overall, disease-specific, disease-free, and progression-free survival with *SIVA1* expression were evaluated using the Kaplan–Meier method. Hazard ratio (HR) and 95% confidence intervals (95% CI) were calculated. *p*-Values indicated in the graphs correspond to the Log-rank test. (b) Analysis of biological processes, cellular components, and molecular functions related to the main differentially expressed genes (DEGs) in comparison to *SIVA1* expression was performed with ShinyGO 0.85.1. All query genes were converted to ENSEMBL gene IDs or STRING-db protein IDs. The false discovery rate (FDR) was calculated based on the nominal *p*-value from the hypergeometric test. FDR threshold = 0.05. (c) Graphs representing the Gene Set Enrichment Analysis results. At the top of the graph, continuous enrichment scores (ES) for the gene set are illustrated. False discovery rate (FDR), nominal *p*-values (NOM *p*-value), and normalized enrichment scores (NES) adjusted for gene set size are indicated. The analysis was performed following the stratification of the breast cancer cohort into its molecular subtypes. (d, e) The volcano plot and Spearman correlation graph were generated using GraphPad Prism 8, illustrating gene sets with statistically significant Log2 Fold Change and *p*-values. (f) The heatmap was constructed with the ClustVis platform, displaying DEGs according to high vs low *SIVA1* expression. *p < 0.05, **p < 0.01, ***p < 0.001. BRCA, Breast Cancer. TCGA, The Cancer Genome Atlas. GO, Gene Ontology.

The TCGA cohort was further subdivided according to histological and molecular subtypes of the disease, as well as to other clinicopathological features; the frequency of patients in these categories crossed with high vs low expression of *SIVA1* is presented in Table 1 and Figure S1b–d. Significant associations were observed between *SIVA1* expression and molecular subtype (*p* = 0.004) as well as histological type (*p* < 0.001). Higher *SIVA1* expression was predominantly detected in the HER2 and basal-like molecular subtypes, as well as in metaplastic and lobular carcinomas (Figure S1b,c).

**Table 1:** Association between *SIVA1* Expression and Molecular and Clinical Factors in the TCGA Breast Cancer Cohort^a^

		*SIVA1*, n (%)		
**clinicopathological factors**	N	low	high	*p*-value
total	1082	541 (50.0)	541 (50.0)	
age				0.201
<60	577	278 (48.2)	299 (51.8)	
≥60	505	263 (52.1)	242 (47.9)	
total	1079	539 (50.0)	540 (50.0)	
primary tumor size (TNM)				0.142
T1 and T2	903	460 (50.9)	443 (49.1)	
T3 and T4	176	79 (44.9)	97 (55.1)	
Total	1062	531 (50.0)	531 (50.0)	
regional lymph node metastasis (TNM)				0.096
N0 and N1	867	444 (51.2)	423 (48.8)	
N2 and N3	195	87 (44.6)	108 (55.4)	
total	915	487 (53.2)	428 (46.8)	
distant metastasis (TNM) TNM)				0.603
M0	894	477 (53.4)	417 (46.6)	
M1	21	10 (47.6)	11 (52.4)	
total	1063	528 (49.7)	535 (50.3)	
TNM stage				0.362
stage I	180	98 (54.4)	82 (45.6)	
stage II	615	307 (49.9)	308 (50.1)	
stage III	249	114 (45.8)	135 (54.2)	
stage IV	19	9 (47.4)	10 (52.6)	
total	945	491 (52.0)	454 (48.0)	
molecular subtype				**0.004**
luminal A/B	696	384 (55.2)	312 (44.8)	
Her2	78	34 (43.6)	44 (56.4)	
basal-like	171	73 (42.7)	98 (57.3)	
total	1081	540 (50.0)	541 (50.0)	
cancer type				**0.000**
invasive ductal carcinoma	780	426 (54.6)	354 (45.4)	
invasive carcinoma	75	38 (50.7)	37 (49.3)	
invasive lobular carcinoma	201	66 (32.8)	135 (67.2)	
mixed mucinous carcinoma	17	9 (52.9)	8 (47.1)	
metaplastic carcinoma	8	1 (12.5)	7 (87.5)	

a TNM: classification system based on primary tumor size (tumor), spread to regional lymph nodes (nodes), and the presence or absence of distant metastasis (metastasis).

Gene ontology investigations indicated that high *SIVA1* expression is associated with increased mitochondrial membrane permeability and ribosome activity. Consistently, induction of DNA repair mechanisms, RNA processing, and spliceosome catalysis were reported (Figure 1b). Elevated *SIVA1* expression and its associated molecular network negatively impacted core cell cycle events, such as chromatin packaging and Golgi complex, centrosome, and cytoskeleton activities. Additionally, there was an attenuation of RNA biosynthesis, catalytic complex assembly, and vesicle-mediated cellular transport (Figure 1b). These functionalities were also highlighted in the gene set enrichment analysis (Figure 1c), together with increased oxidative phosphorylation. The stratification of the cohort into its molecular subtypes revealed, through Gene Set Enrichment Analysis (GSEA), a negative association between gene expression and the mitotic spindle pathway in the Luminal A/B and Basal-like groups, as well as between *SIVA1* and TGF-β signaling in the HER2 subtype.

Differential gene expression profile (Figure 1d) and Spearman coefficients (Figure 1e) emphasized that genes negatively correlated with *SIVA1* expression, such as *TOR1AIP2*, *RB1CC1*, *UBR5*, *CEP350*, *BROX*, *ATF1*, *ASH1L*, *UHMK1*, *CFAP97*, *DENND4A*, *APC*, *BIRC6*, and *MAP3K2*. Genes that described a positive correlation included *GUK1*, *C1orf35*, *XRCC3*, *UBALD1*, *BOLA1*, *PACS2*, *ZNF205*, *PAXX*, *TRMT61A*, *BRF1*, and *NUDT14*. These results underscore *SIVA1*’s influence on DNA repair processes, RNA methylation, RNA polymerase III function, and mitochondrial respiratory chain formation. Conversely, negatively correlated genes are involved in vesicular trafficking, cell cycle regulation, apoptosis inhibition, and other signaling cascades such as MAPK. These gene associations remain consistent within the analyzed cohort, as illustrated by the heatmap (Figure 1f).

### 2.2 SIVA1 Is Broadly Expressed across Breast Cancer Cell Lines, and Triple-Negative Knockdown Models Show Increased Dividing Rate and PTX Sensitivity

Protein levels of SIVA1 were measured in nontumorigenic and tumor breast cell lines, revealing broad expression across cell types and higher abundance in the triple-negative cell lines MDA-MB-231, BT-549, and HCC1937, as well as in the luminal A cell line MCF-7 (Figure 2b). Given the clinical relevance of triple-negative breast cancer, for comparative purposes, a high (BT-549) and a low (Hs578T) *SIVA1*-expressing cell line were selected for gene knockdown via shRNA (short hairpin RNA). This approach enabled the evaluation of whether the presence of the protein, even at low levels, is sufficient to induce a significant cellular adaptation. Western blotting results (Figure 2c) showed a significant reduction in protein levels in the *SIVA1* shRNA models for both lineages (*p* < 0.05), a result corroborated by quantitative PCR (qPCR) (*p* < 0.0001) (Figure 2d).

**Figure 2:**
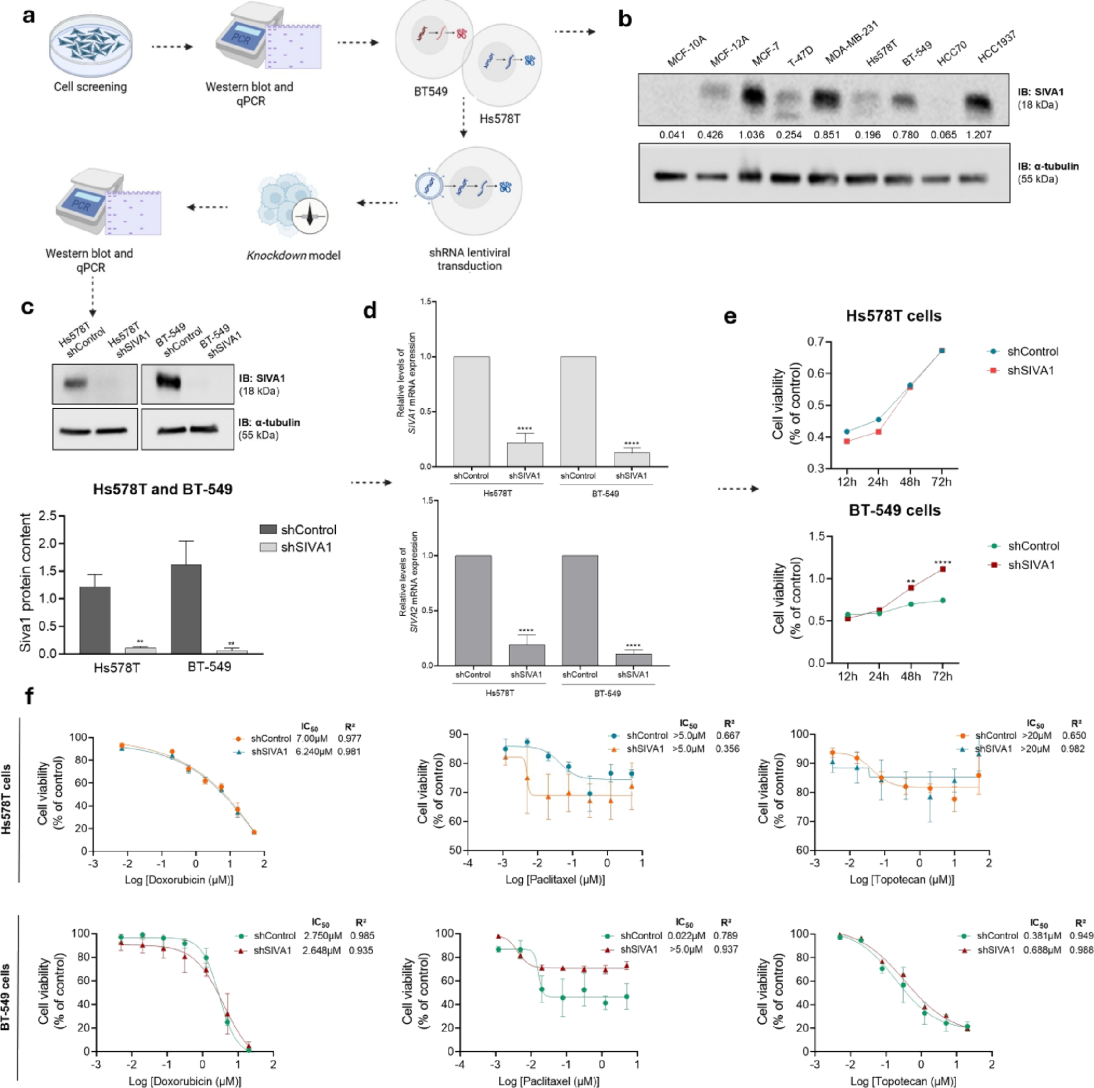
SIVA1 has broad expression across breast cancer cell lines, with gene knockdown linked to increased division rates and sensitivity to taxanes in triple-negative models. (a) Workflow of the testing and knockdown procedures with shRNA in breast cancer cells. (b) Western blotting analysis of SIVA1 in total extracts from tumor and nontumor breast cell lines. Membranes were reprobed with anti-α-tubulin antibody for loading control and normalization, and detection was performed using SuperSignal West Dura Extended Duration Substrate and the G:BOX system. (c, d) Validation of *SIVA1* knockdown by lentiviral shRNA transduction via Western blotting and qPCR. (e) Following model confirmation, the role of *SIVA1* in cell growth rates was initially assessed by the MTT reduction assay. (f) Cell models were exposed to DOX, PTX, and TPT for 72 h to assess viability, also by MTT assay. *p*-Values for shControl vs. shSIVA1 analyses at each curve point are indicated (*n* = 3). *p < 0.05, **p < 0.01, ***p < 0.001, ****p < 0.0001; Student’s *t*-test. DOX, doxorubicin; PTX, paclitaxel; TPT, topotecan.

After model validation, the impact of gene knockdown on tumor cell growth was assessed (Figure 2e). For the higher-expressing cell line (BT-549), knockdown cells exhibited increased growth capacity compared to the control group (*p* < 0.0001). Complementing these findings, MTT assays following treatment with doxorubicin (DOX), paclitaxel (PTX), and topotecan (TPT) were conducted. Among the tested drugs, shSIVA1 cells exhibited an altered response only to PTX. In this context, the data suggest that in Hs578T cells with *SIVA1* knockdown, PTX showed greater efficacy compared to the control. Conversely, in BT-549 knockdown cells, the opposite pattern was observed, with PTX showing reduced efficacy in knockdown cells. However, neither cell line reached complete inhibition, with IC_50_ values exceeding 5 μM. The viability response to the other compounds remained similar between shSIVA1 and shControl cultures, as detailed in Figure 2 **f.**

### 2.3 DOX and PTX Treatments Do Not Alter SIVA1 Cellular Localization in Breast Cancer Cells

Addressing the knowledge gap regarding SIVA1 intracellular translocation, immunofluorescence assays were performed following treatment with DOX and PTX. Both control and treated groups exhibited cytoplasmic and nuclear localization of SIVA1, with the protein accumulating especially in the nucleus in punctate patterns (Figure 3). No morphological changes were detected in the knockdown models (Figure S2).

**Figure 3:**
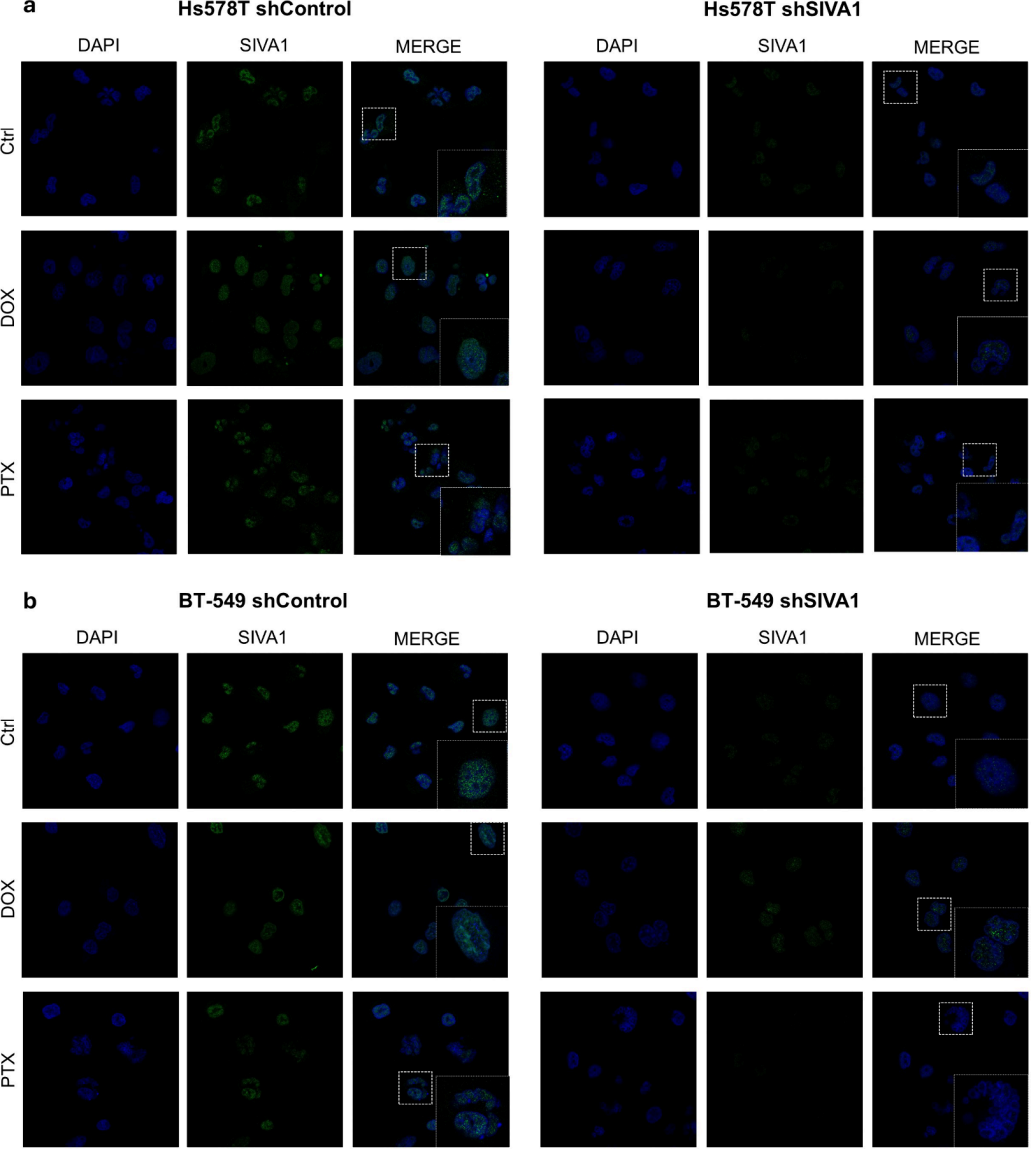
SIVA1 displays predominantly nuclear punctate localization in breast cancer cell lines, independent of treatments. (a, b) Cell models were treated with DOX and PTX for 24 h, followed by fixation, permeabilization, and staining with anti-Siva (1:100) and anti-rabbit Alexa Fluor 488 (1:600). In the figure, SIVA1 is shown in green, and nuclei in blue (DAPI staining), with the MERGE representing the overlay of all three signals. Images were taken in the confocal microscope Stellaris-WLL. Images were acquired using a 20× objective.

### 2.4 *SIVA1* Knockdown Modifies Cell Cycle-Related Responses and Colony Formation under PTX Treatment in Hs578T and BT-549 Cell Lines

PTX was selected for subsequent experimental analyses due to its greater difference in response between knockdown and control cell lines. Measurement of apoptosis rates in treated cells reinforced the drug’s efficacy in inducing cell death in both models (*p* < 0.0001) (Figure 4a,b). A statistically significant difference in basal apoptosis rates (untreated groups) was observed between shRNA cells, with shSIVA1 showing an increased apoptosis rate (*p* < 0.05).

**Figure 4:**
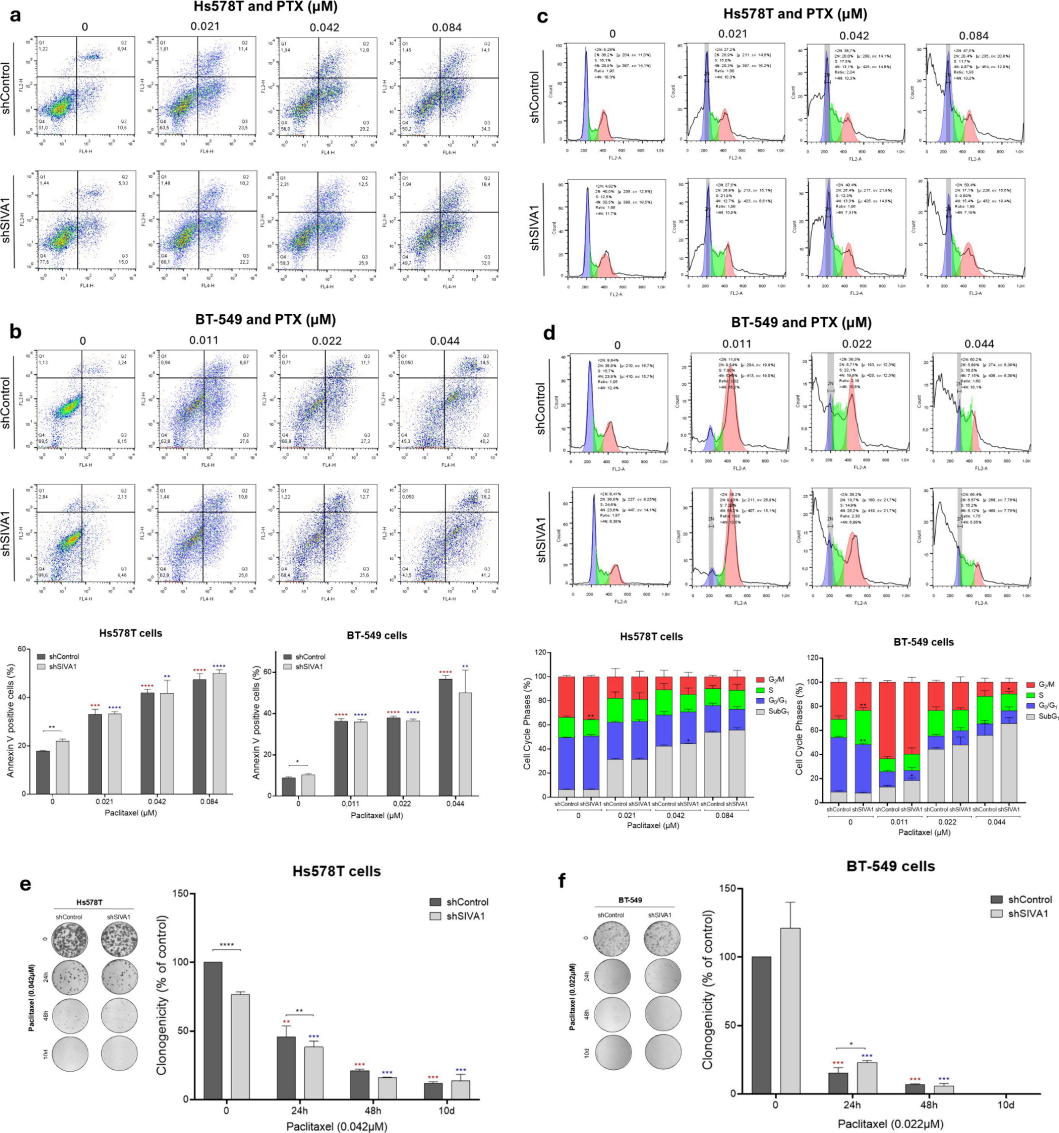
Impact of *SIVA1* knockdown in the cell cycle control and clonogenic capacity of Hs578T and BT-549 cells. (a, b) Transduced cells were treated with vehicle or PTX for 48 h. Representative dot plots were constructed for each condition, illustrating Annexin V–positive cells in the upper and lower right quadrants. Bar graphs represent the mean ± SD of at least three independent experiments. (c, d) Different phases of the cell cycle were distinguished based on propidium iodide (PI) staining after 24 h of PTX treatment. Quantification of SubG_1_, G_0_/G_1_, S, and G_2_/M populations is shown in histograms and bar graphs (*n* = 3). (e, f) Colonies containing viable cells were fixed with ethanol and detected by 0.1% crystal violet in 10% ethanol staining after 1, 2, or 10 days of treatment with PTX or vehicle. Representative images are placed to the left of the bar graphs, which present mean ± SD (*n* = 3). *p*-Values for control vs. treated groups (*shControl in red; *shSIVA1 in blue), and shControl vs. shSIVA1 (*in black) for each condition are indicated. *p < 0.05, **p < 0.01, ***p < 0.001, ****p < 0.0001; ANOVA and Bonferroni post hoc test.

Given the recognized role of *SIVA1* in cell cycle regulation, further investigations were designed to analyze its impact on phase transition in cultures treated with PTX. For both cell lines (Figure 4c,d), an increase in the SubG_1_ population of the knockdown-treated group was observed (*p* < 0.05), although with a small effect in Hs578T cells. It is noteworthy that the first dose of treatment led to G_2_/M arrest in the BT-549 cell line, a result that reinforces the compound’s effectiveness.

Consistent with apoptosis findings, clonogenic capacity was reduced following the compound application (Figure 4e,f). Clonal growth exhibited time-dependent behavior, with a drastic reduction in colony number starting from 24 h of treatment. In the BT-549 cell line, PTX completely inhibited clonal growth rate by day 10 (*p* < 0.0001). For Hs578T cells, it was reduced by 82–88% (*p* < 0.0001). Quantitatively, colony numbers were lower in Hs578T knockdown cells compared to control cells, both in untreated conditions (*p* < 0.0001) and under 24 h of drug exposure (*p* = 0.003). For BT-549, the knockdown model exhibited increased clonogenicity, particularly under 24 h of treatment (*p* = 0.036). From the 24 h time point onward, the effect of PTX on colony formation was independent of *SIVA1* expression levels.

### 2.5 *SIVA1* Knockdown Spheroids Exhibit Increased Sensitivity to PTX Treatments

To mimic the structural and spatial organization of tumors, spheroids were generated. *SIVA1* knockdown enhanced the effects of PTX treatment, resulting in smaller spheroids in Hs578T knockdown cells upon increasing treatment concentrations (Figure 5a–d) and in spheroids with a higher disintegration rate following drug exposure in BT-549 knockdown cells (Figure 5b–e). In both cellular models, untreated knockdown cells exhibited a smaller diameter compared to shControl cells, an effect that was more pronounced in the BT-549 cell line (Figure 5c). In the Hs578T line, a reduction in ATP production (*p* = 0.043 at 0.168 μM) was observed in shSIVA1 compared with shControl at the highest PTX dose (Figure 5f). For BT-549 knockdown cultures, results also showed decreased ATP production (*p* = 0.004 at 0.176 μM) in knockdown cells (Figure 5f). Propidium iodide (PI) staining revealed an increase in labeled cells within the necrotic core in Hs578T knockdown (Figure 5d) and around the spheroid periphery in BT-549 knockdown spheroids (Figure 5e) following PTX treatment, indicating enhanced cell death in *SIVA1* knockdown cells.

**Figure 5:**
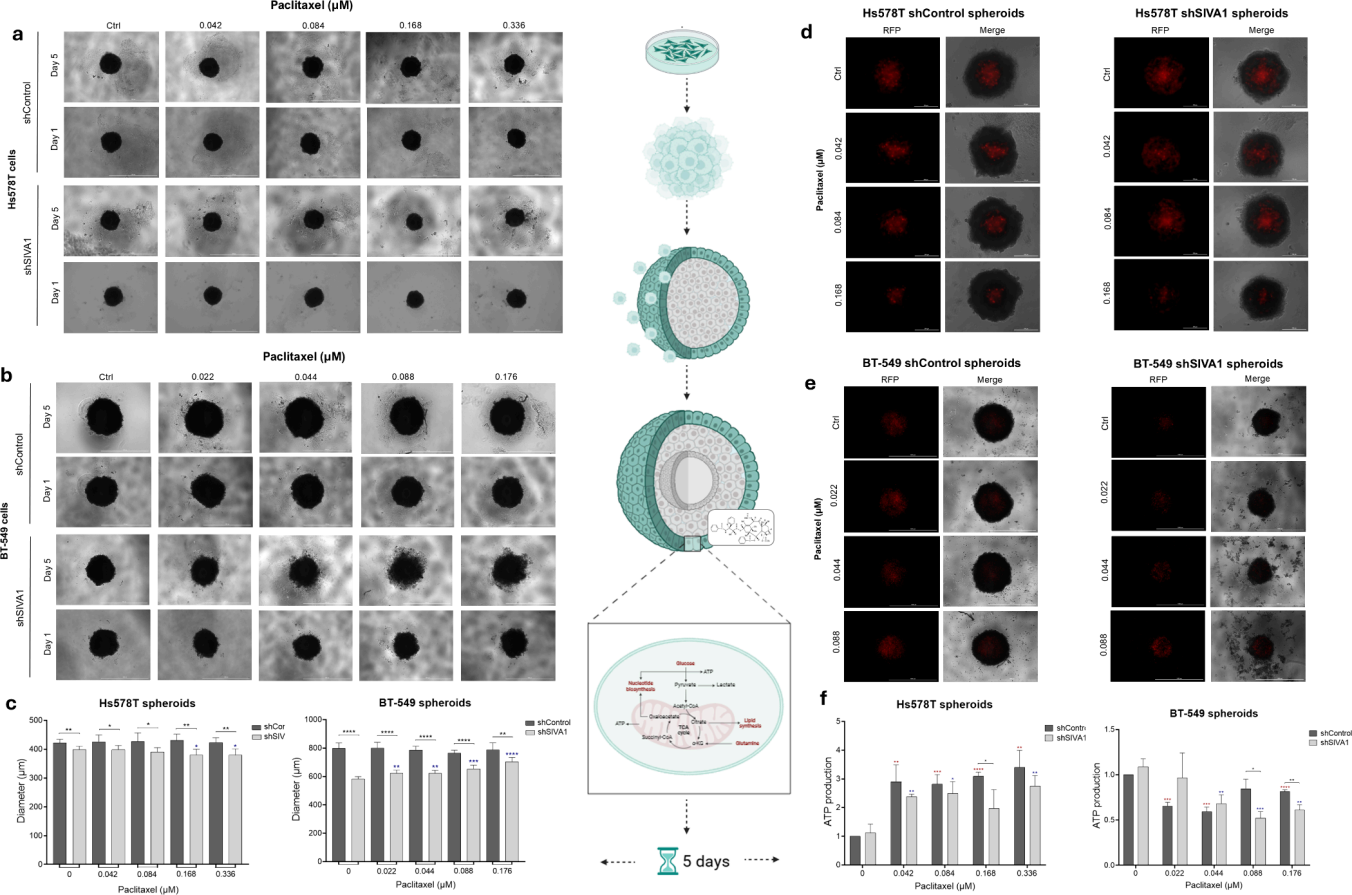
*SIVA1* knockdown results in reduced spheroid cohesion and greater impact of PTX treatments on cellular metabolism and viability. (a, b) Three-dimensional cultures were generated by growing cells on 1% agarose-coated plates, maintained for 5 days under PTX treatment. (c) Bar graphs represent mean ± SD of spheroid diameters (*n* = 5). (d, e) Five samples from each condition were selected for ATP production measurement on day 5 and for PI staining. The MERGE image shows the overlay of phase contrast and PI staining. (f) Bar graphs represent mean ATP levels for the four cell lines. *p*-Values for control vs. treated groups (*shControl in red; *shSIVA1 in blue), and shControl vs. shSIVA1 (*in black) for each condition are indicated. *p < 0.05, **p < 0.01, ***p < 0.001, ****p < 0.0001; ANOVA and Bonferroni post hoc test. Scale bar = 1000 μm.

### 2.6 *SIVA1* Expression Influences Cellular Migration and Invasion Processes, Ubiquitination, and the Stathmin 1 and P53 Pathways in Breast Cancer

Migration assays reinforced the role of SIVA1 in regulating this process, with *SIVA1* knockdown BT-549 cells displaying enhanced migratory capacity (*p* < 0.0001) (Figure 6a). Similar results were obtained in invasion assays (Figure 6b), where *SIVA1* knockdown correlated with increased invaded areas in both cell lines (*p* < 0.05).

**Figure 6:**
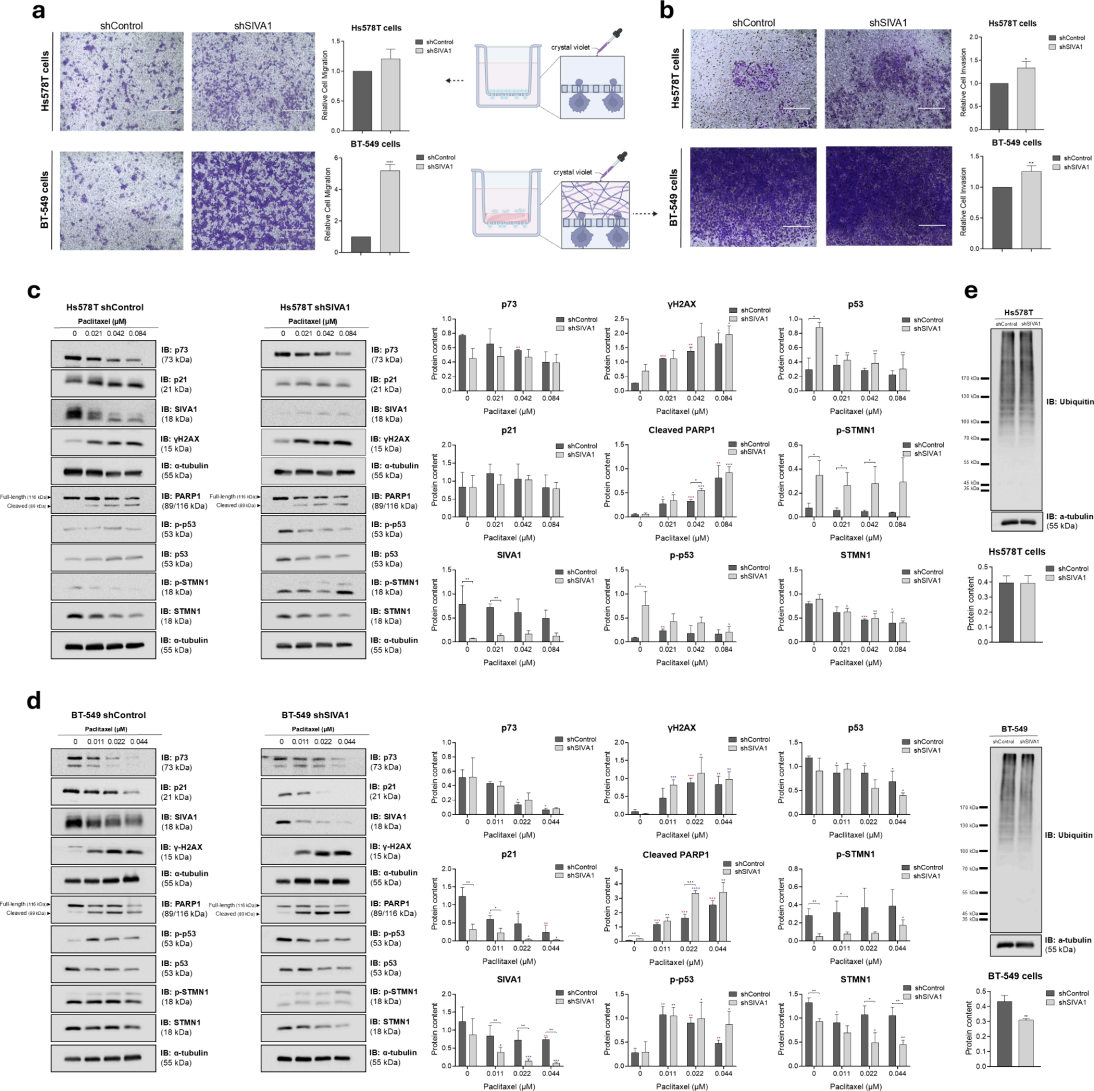
*SIVA1* knockdown breast cancer cells exhibit increased migratory and invasive capacity, with reduced ubiquitination and enhanced activation of STMN1 and p53 pathways. (a) The *SIVA1* knockdown and control Hs578T and BT-549 were cultured in Transwell plates containing polycarbonate membranes with 8 μm pores for 36 h, followed by fixation, staining with 0.1% crystal violet in 10% ethanol, and counting of cells in seven random fields per well. Images were captured using the EVOS AME-3302 microscope (*n* = 3). (b) Cells were also cultured in plates with inserts containing a Matrigel layer (1:5 in BFS) for 48 h, and the average invaded area per image and condition was calculated (*n* = 3). (c–e) Western blotting analyses of p73, p21, SIVA1, γH2AX, PARP1, phospho­(p)-p53, p53, phospho­(p)-STMN1^S16^, STMN1, and ubiquitin in total extracts from cells treated with PTX or vehicle for 24 h. Membranes were incubated with the indicated antibodies and developed using the SuperSignal West Dura Extended Duration Substrate system on the G:BOX Chemi XRQ equipment. α-tubulin was used as a loading control. Bar graphs represent mean ± SD (*n* = 3). *p*-Values comparing shControl vs. shSIVA1 groups are indicated. *p < 0.05, **p < 0.01, ***p < 0.001; Student’s *t*-test.

Supporting the immunofluorescence data, Western blotting analyses indicated a modulation of SIVA1 protein levels by PTX treatment, showing a dose-dependent decrease (Figure 6c,d). Both knockdown cell lines exhibited higher levels of cleaved PARP1 (*p* < 0.05) after drug addiction. Reduced expression of STMN1 and its phosphorylated inhibited form was observed in BT-549 shSIVA1 models (*p* < 0.05). Hs578T knockdown cells showed increased p53 (*p* = 0.004), phosphorylated p53 (p-p53) (*p* = 0.015), and p-STMN1 (*p* = 0.023) content in the untreated condition compared to controls. Lastly, analysis of protein ubiquitination revealed a significantly decreased ubiquitination rate in BT-549 cells transduced with shSIVA1 (*p* = 0.007) (Figure 6e).

Differential expression of *SIVA1* was observed between tumor and normal breast tissues, with its overexpression representing a characteristic feature of samples from diagnosed patients. Its involvement in breast cancer development has also been supported by previous studies, which have linked germline variation in SIVA1 to disease incidence across age groups.^12^

Although no significant association was found between the *SIVA1* expression and patient survival in the TCGA cohort, this does not preclude its biological relevance. Variations across histological types and molecular subtypes, also reported in other studies,^19,25^ suggest an association with tumor-specific features. However, analyses based on bulk tumor data may obscure its expression in malignant cells,^32^ while mRNA levels do not necessarily reflect the abundance of the active protein due to post-transcriptional regulation.^33^ Moreover, *SIVA1* likely operates within complex signaling networks, and its clinical impact may depend on interactions with additional molecular factors,^34^ underscoring the need to validate *in silico* findings in *in vitro* models.

Careful investigation of the SIVA1 protein content and gene expression signaled its prominent presence in breast tumor cell lines. Cross-referenced with bioinformatics data, higher protein levels were confirmed in some TNBCs, a classification recognized as more aggressive among the subtypes.^5^ The positive regulation of SIVA1 protein content in tumor models may arise as a response to stress,^13^ ongoing DNA damage,^12^ or excessive cellular proliferation.^13,35^

Returning to its central role as a gene regulator, an immunofluorescence assay of tumor cell lines revealed the presence of the protein in both cytoplasmic and nuclear compartments, with predominant localization in the nucleus. This finding aligns with the investigations by Py et al. (2004),^29^ which predict the involvement of the SIVA1 N-terminal domain in their translocation to the nucleus. Additionally, colocalization assays with multiple organelle markers supported its correlation with endocytic vesicles and the cytoskeleton,^14^ consistent with some functions revealed by bioinformatics analyses.

Among these functions enriched in samples from patients with high *SIVA1* expression, the response to DNA damage, RNA splicing or methylation, ubiquitination, and mitochondrial membrane permeabilization it was identified. Thus, this consolidates literature data that indicates the SIVA1-p53 and SIVA1-MDM2 interactions as important regulatory pathways of the repair system,^12,36,37^ as well as SIVA1’s interaction with death receptors^13^ and other apoptosis-related proteins,^29,38^ supporting its pro-apoptotic role. Additionally, reduced *SIVA1* expression appears to favor cell cycle progression and RNA synthesis, preparing the cell for cytokinesis. The effects of *SIVA1* on cytoskeletal dynamics were clarified by Zhang et al. (2014),^39^ who described its role as a microtubule stabilizer and focal adhesion formation blocker in HCT116 cell lines.

To validate bioinformatic findings, knockdown cell lines were generated via lentiviral shRNA transduction, a method previously applied in other studies.^16,40,41^ Initial tests demonstrated higher growth rates in the BT-549 knockdown line compared to the control, reaffirming SIVA1’s relevance in regulating tumor growth.^36,42,43^ Treatment with DNA-damaging agents (DOX and TPT) and a microtubule stabilizer (PTX) exposed a differential sensitivity of knockdown models only to PTX. Therefore, it is suggested that *SIVA1*’s primary effect in TNBC cellular models lies in cytoskeletal maintenance.

Although *in silico* analyses implicated SIVA1 in the DNA damage response, our *in vitro* experiments did not fully corroborate this prediction, since knockdown and control cells exhibited comparable sensitivities to doxorubicin and topotecan. This discrepancy suggests that SIVA1’s contribution to DNA damage signaling is context-dependent, potentially facilitating repair under moderate stress,^11^ an effect that may be masked under intense genotoxic conditions as simulated in the treatments presented. Moreover, *in vitro* models may not fully recapitulate the complexity of tumor biology, including microenvironmental influences and cumulative genomic stress *in vivo*.^44^

In this context, the literature shows that SIVA1, maintaining dual functionality as a DNA damage repairer and a cytoskeletal stability regulator, translocates from the cytoplasm to the nucleus under certain conditions,^24,45,46^ though the stimuli required remain unclear. Immunofluorescence after DOX and PTX treatment evidenced protein abundance in the nuclear region regardless of drug or dose, with no major changes in the cytoplasmic/nucleus accumulation and no translocation for DNA repair after drug exposure.

Beyond its interaction with death-inducing proteins,^21^ SIVA1 cytoplasmic presence is essential for CaMKII/STMN1 complex formation and subsequent STMN1 phosphorylation and inactivation.^47^ Consistent with recent studies indicating STMN1 as important for TNBC proliferation,^48^ cells with predominance of its active form (BT-549 knockdown cell line) showed higher growth rates. Increased growth may explain their greater susceptibility to the well-established role of PTX as a mitotic inhibitor,^49–51^ as a larger fraction of cells is actively undergoing division.

Regarding PTX response, basal apoptosis rates were higher in Hs578T and BT-549 shSIVA1 cells without significant differences between treatments. This variability may relate to SIVA1’s role in MDM2/p53 interaction and subsequent p53 degradation. Lower SIVA1 is expected to increase p53 activity, enhancing its tumor suppressor function,^52^ even in untreated cells. Lack of difference between shControl and shSIVA1 responses at tested concentrations may be partially explained by PTX effects being independent of p53 status.^53,54^ The knockdown cell lines’ increased sensitivity to taxanes was reinforced by cell cycle analysis, revealing increased SubG_1_ populations in knockdown models. SIVA1’s role in cell cycle progression was also reported by Kong et al. (2023)^35^ in gastric cancer cells. Moreover, PTX effectively reduced colony numbers in a time-dependent manner, with the increased clonogenicity observed in the BT-549 shSIVA1 model after 24 h of treatment possibly resulting from an elevated proliferation rate.

Accordingly, spheroids derived from Hs578T knockdown cell lines exhibited a marked reduction in diameter at higher drug concentrations, whereas BT-549 spheroids showed an apparent increase because of the disaggregation of their inner zones. The spheroid architecture generates nutrient and diffusion gradients that more closely resemble the tumor microenvironment and drug penetration observed *in vivo*. Consequently, spheroids serve as a relevant intermediate model between conventional monolayer cultures and animal studies, enabling preliminary compound screening while helping to reduce animal use.^55^

Collectively, these results indicate alterations in cell adhesion properties. Junction weakening likely arises from increased active STMN1 in shSIVA1 cells and induced microtubule depolymerization, destabilizing adhesions and contributing to enhanced migration, as detailed by other groups.^48,56–58^ Decreased ATP production and increased PI-positive in BT-549 shSIVA1 cells indicate greater compound potency in this line. Since ATP levels serve as an indirect measure of viability,^59^ combining this assay with PI staining revealed a similar effect in Hs578T spheroids, reflected by loosening proliferative zones, reduced spheroid compactness, and a necrotic zone, and increased PI-positive cells.

The proposed coparticipation of SIVA1 in reducing adhesion expression via STMN1 ^47^ is supported by migration and invasion assays, where knockdown cells showed significantly increased migratory capacity, aligning with Kong et al.'s (2023),^35^ Machado-Neto et al.'s (2015),^41^ Chen et al.'s (2015),^36^ and Li et al.'s (2011)^47^ findings. As discussed previously, SIVA1-mediated cytoskeletal dynamics interference would prevent epithelial-mesenchymal transition (EMT), migration, and extracellular matrix invasion,^27,60^ as reproduced in these assays.

Reduced inactive STMN1 in knockdown models was confirmed by Western blotting, along with increased p53 active form. These results support previous studies,^11,36,61^ tracing the SIVA1-p53 interaction as crucial for MDM2 binding and its activity. Phosphorylated p53 executes cell cycle arrest and apoptosis induction,^21^ effects seen in cell cycle and viability assays. Regarding treatment efficacy, an increase in cleaved PARP1 was reported at higher doses, suggesting a PTX-induced DNA damage and apoptosis^62,63^ especially in knockdown models. SIVA1’s relevance in protein ubiquitination was demonstrated in BT-549 cells, where its content positively correlated with pan-ubiquitin labeling. Some of its known ubiquitination targets are ARF,^36^ PCNA,^40^ and AKT1.^64^ Altogether, decreased ubiquitination can stabilize key regulatory proteins, leading to p-p53 accumulation and increased active (dephosphorylated) STMN1, which promotes cell cycle arrest, apoptosis, and altered microtubule dynamics.

The data presented herein substantiate the critical role of SIVA1 in breast cancer, particularly within triple-negative subtypes, which are characterized by pronounced aggressiveness and a limited repertoire of actionable therapeutic targets. In addition to implicating SIVA1 in the preservation of cellular architecture and the modulation of the response to taxane-based therapies, these findings provide a compelling rationale for its evaluation as a biomarker for patient stratification. Elevated *SIVA1* expression in breast tumors may guide therapeutic strategies toward overcoming drug resistance,^65,66^ whereas reduced expression levels may highlight the need for interventions aimed at curbing the proliferative and metastatic potential of neoplastic cells.

## 3 Conclusions

In conclusion, the key findings include increased cell growth, migration, and invasion following SIVA1 reduction in breast cancer cell lines, suggesting that this protein primarily functions as a regulator of cytoskeletal dynamics. Its impact on spheroid diameter suggests involvement in adhesion maintenance, hindering EMT,^25,39,67^ thus acting as a protective factor against malignancy progression. Differential PTX treatment effects likely derive from *SIVA1*’s influence on STMN1. *SIVA1* expression may serve as a predictive biomarker for taxane response, enabling patient stratification and more personalized therapies. High expression suggests the need to overcome drug resistance, while low levels indicate targeting tumor proliferation and metastasis. *In vivo* preclinical validation is essential for clinical translation.

## 4 Material and Methods

### 4.1 Cell Lines and Compounds

The MCF-10A, MDA-MB-231, BT-549, HCC70, and HCC1937 cell lines were kindly provided by Dr. Silvina Odete Bustos (Instituto do Cancer do Estado de São Paulo, Octavio Farias de Oliveira, São Paulo, Brazil), while the MCF-12A, MCF-7, T-47D, and Hs578T cell lines were obtained from Prof. Glaucia Maria Machado Santelli (Institute of Biomedical Sciences, São Paulo, Brazil). Cell culture conditions were maintained according to the DSMZ (German Collection of Microorganisms and Cell Cultures) recommendations, and the cultures were routinely tested for mycoplasma contamination using DAPI (4′,6-diamidino-2-phenylindole) staining. The culture media used were DMEM-F12 (Dulbecco’s modified Eagle’s medium) with 10% fetal bovine serum (FBS) for MDA-MB-231, Hs578T, and MCF-7 cell lines; RPMI with 10% FBS for T-47D, HCC1937, HCC70, and BT-549 lines; and phenol red-free DMEM-F12 supplemented with 5% horse serum, 1% penicillin/streptomycin, 0.02 μg/mL EGF, 0.5 μg/mL hydrocortisone, 0.1 μg/mL cholera toxin, and 10 μg/mL insulin for MCF-10A and MCF-12A lines. The histological type and molecular subtype of each cell line are specified in Table S1. DOX (#D1515), PTX (#PHR1803), and TPT (#T2705) were purchased from Sigma-Aldrich (St. Louis, MO, USA) and were diluted and stored according to the manufacturer’s instructions. Drug selection was guided by compounds widely employed in contemporary breast cancer treatment regimens.^17,45,68^

### 4.2 Lentiviral Transduction with shRNA

Parental Hs578T and BT-549 cell lines were transduced with lentiviral particles encoding either *SIVA1*-targeting short hairpin RNA (shSIVA1; Santa Cruz, sc-37385-V) or a nontargeting control shRNA (shControl; Santa Cruz, sc-108080), both provided at a titer of 5 × 10^6^ IFU/mL. Cells were seeded in 12-well plates at a density of 1 × 10^4^ cells per well. After 24 h, 10 μL of the respective lentivirus and 6 μg/mL Polybrene were added to each well (multiplicity of infection: 5). Following a 24 h incubation period, puromycin was added at a final concentration of 1 μg/mL to select for successfully transduced cells. Stable clones were subsequently validated for *SIVA1* knockdown by Western blotting and qPCR and expanded for downstream analyses.

### 4.3 MTT Assay

A total of 1 × 10^4^ Hs578T or BT-549 cells stably expressing shControl or shSIVA1 were seeded per well in 96-well plates and cultured in the appropriate medium supplemented with 10% FBS. Cells were then exposed to increasing concentrations (0.0012–5 μM) of PTX, DOX, or TPT for 72 h. Following treatment, 10 μL of MTT solution (5 mg/mL) was added to each well, and plates were incubated at 37 °C for 4 h. The reaction was stopped by adding 100 μL of 0.1 M HCl in anhydrous isopropanol, and absorbance was measured at 570 nm using a SpectraMax M5 plate reader (Molecular Devices). The half-maximal inhibitory concentration (IC_50_) was estimated by nonlinear regression using a four-parameter logistic model (log­[inhibitor] vs response, variable slope) in GraphPad Prism. Model parameters included bottom, top, logIC_50_, and hill slope. IC_50_ values were derived from the fitted curves, with goodness of fit assessed by *R* ^2^ and 95% confidence intervals obtained by profile likelihood analysis. Additionally, this assay was used to generate growth curves of the transduced cell lines under untreated conditions, at 12, 24, 48, and 72 h.

### 4.4 Clonogenic Assay

For the colony formation assay, Hs578T and BT-549 cells (shControl or shSIVA1) were seeded at a density of 1 × 10^3^ cells/mL in 6-well plates and exposed to either vehicle or PTX at concentrations of 0.042 μM (Hs578T) and 0.022 μM (BT-549). The plates were incubated at 37 °C, 5% CO_2_, and assessments were carried out after 24 h, 48 h, or 10 days of treatment. Colonies were detected by adding 1 mL of 0.1% crystal violet in 10% ethanol, then documented using the G:BOX Chemi XRQ system (Syngene). Colony counting was performed using ImageJ software (US National Institutes of Health).

### 4.5 Apoptosis Assessment

Hs578T and BT-549 cells (shControl or shSIVA1) were seeded at a density of 1 × 10^5^ cells/well in 6-well plates and treated with vehicle or PTX in the ranges of 0.021–0.084 μM (Hs578T) and 0.011–0.044 μM (BT-549). Cells were incubated for 48 h, then washed twice with cold phosphate-buffered saline (PBS) and resuspended in a binding buffer containing 1 μg/mL PI and 1 μg/mL annexin V-APC. Samples were incubated for 15 min at room temperature, protected from light, followed by flow cytometry analysis using a FACSCalibur system (Becton Dickinson). Data was analyzed with FlowJo software (Treestar).

### 4.6 Cell Cycle Analysis

The control and knockdown models were seeded at a density of 1 × 10^5^ cells per 100 mm Petri dish and treated with 0.021–0.084 μM (Hs578T) or 0.011–0.044 μM (BT-549) of PTX for 24 h. Cells were washed with PBS and resuspended in 100 μL PBS, followed by the addition of 75% ethanol (900 μL) and temporary storage on ice. After centrifugation at 1200 rpm for 5 min, the supernatant was discarded, and the cells were washed again with PBS. The pellet was resuspended in 300 μL of staining solution containing 0.1% Triton X-100, 0.1 mg/mL RNase (Merck), and 1 μg/mL PI (Sigma-Aldrich). Cells were incubated for 30 min, protected from light, and analyzed on a BD FACSCalibur flow cytometer, acquiring 10,000 events per condition.

### 4.7 Protein Extraction and Western Blotting

Total proteins were extracted using a buffer containing 100 mM Tris (pH 7.6), 1% Triton X-100, 2 mM PMSF, 10 mM Na_3_VO_4_, 100 mM NaF, 10 mM Na_4_P_2_O_7_, and 10 mM EDTA. Equal amounts of total protein (30 μg) were subjected to SDS-PAGE and Western blotting with antibodies listed in Table S2. Signal detection used SuperSignal West Dura Extended Duration Substrate (Thermo Fisher Scientific) and Gel Doc XR+ system (Bio-Rad). Images were captured using the G:BOX Chemi system (SynGene) and quantified with UN-SCAN-IT gel 6.1 software (Silk Scientific).

### 4.8 Quantitative PCR (qPCR)

Samples containing 1 μg of RNA from breast cancer cell lines were reverse transcribed into cDNA using the High-Capacity cDNA Archive Kit (Thermo Fisher Scientific). Gene amplification was performed on a QuantStudio 3 Real-Time PCR System using Power SYBR Green PCR Master Mix (Thermo Fisher Scientific). Sixty nanograms of each cDNA sample were used in triplicate, and a negative control (RNase-free water replacing cDNA) was included for each primer pair. The reference genes were *HPRT1* and *ACTB*, and relative expression was calculated using the 2^–ΔΔCT^ method.^69^ The primers are listed in Table S3.

### 4.9 2D Cell Culture Migration and Invasion Assays

Migration and invasion capacities of control and *SIVA1* knockdown models were evaluated using Transwell systems with polycarbonate membranes and 8 μm pores. For the invasion assay, 2 × 10^5^ cells/well were seeded in 24-well inserts (Millipore, #MCEP24H48), precoated with 50 μL of Matrigel (Corning, #354237) diluted 1:5 in 0.1% FBS medium. For the migration assay, 1 × 10^5^ cells/well were seeded directly in inserts (Nest, #NST-725301) without Matrigel. In both assays, cells were resuspended in serum-free medium and added to the upper chamber, while the bottom chamber contained complete medium (10% FBS) as a chemoattractant. Incubation was carried out at 37 °C for 48 h (invasion) or 36 h (migration). Following incubation, membranes were washed with 1X PBS, fixed in cold 100% methanol for 10 min, and stained with 0.1% crystal violet in 10% ethanol for 15 min. Inserts were rinsed, dried, and imaged using an EVOS AME-3302 microscope at 10× magnification. For each condition and cell line, three independent experiments were conducted, with seven random fields imaged per insert. ImageJ was used for the analysis of the total invaded area and the number of migrated cells.

### 4.10 3D Cell Culture Formation and Viability Assays

Spheroids were formed in 96-well plates coated with 65 μL of 1% agarose per well. Cells from the Hs578T and BT-549 (shControl or shSIVA1) lines were seeded at densities of 1 × 10^4^ and 2 × 10^4^ cells per well, respectively, and maintained at 37 °C. After 3 days, treatment with 0.042 μM (Hs578T) and 0.022 μM (BT-549) of PTX was carried out, and cells were further maintained for 5 days. Spheroid formation was recorded using images captured with the LionHeart FX Automated Microscope (BioTek Instruments) on days 1, 3, and 5, starting from the compound addition. For comparison purposes, 5 spheroids from each condition were selected to measure their respective diameters on ImageJ Software and for PI (10 μg/mL) staining on the last day of treatment. Complementary to the statistical analysis of diameters and PI staining, ATP production was assessed with CellTiter-Glo 3D Cell Viability Assay reagent (Promega, #G9681; 100 μL/well). The white 96-well plates containing the spheroids were shaken on a horizontal shaker at 120 rpm for 5 min and incubated at room temperature for 25 min to stabilize the luminescent signal. Readings were performed using a luminometer with WinPro software (integration time ranging from 0.25 to 1 s).

### 4.11 Immunofluorescence Microscopy

The shRNA-transduced cell lines were seeded onto glass coverslips (Knittel glass), treated with PTX (0.042 μM for Hs578T and 0.022 μM for BT-549) and DOX (7 μM for Hs578T and 2.75 μM for BT-549), washed with PBS, and fixed with 4% formaldehyde solution. Subsequently, permeabilization was performed using 0.5% Triton, followed by blocking with 3% bovine serum albumin (BSA) solution. Anti-Siva (Thermo Fisher Scientific, #PA5–100737; 1:100) and anti-Rabbit Alexa Fluor 488 (Thermo Fisher Scientific, #A-11034; 1:600) antibodies were applied. Plates with primary antibody were incubated in a humid chamber at 4 °C for 24 h. After this period, wells were washed 3 times with PBS, incubated for 1 h with the secondary antibody, and washed again with PBS. Finally, coverslips were mounted on slides with fluorescence mounting medium (ProLong Gold with DAPI, Life Technologies) for visualization on the Stellaris-WLL confocal microscope, using a 20× objective for image acquisition.

### 4.12 Gene Set Enrichment Analysis (GSEA)

Clinical and gene expression data were collected from the cBioPortal platform (https://www.cbioportal.org/), selecting the Invasive Breast Carcinoma TCGA PanCancer Atlas cohort (n = 1082) as the reference. Molecular interaction investigations were performed using Gene Set Enrichment Analysis (GSEA v.4.0; Broad Institute), measuring correlations based on normalized enrichment score (NES), false discovery rate (FDR), and *p*-values, defined from gene sets curated by MSigDB Hallmarks, Reactome, and KEGG. Enrichment scores (ESs) were calculated using the Kolmogorov–Smirnov statistic, tested for significance by 1000 permutations, and normalized (NES) to adjust for gene set size. An FDR *q*-value cutoff below 0.25 was set for significance.

### 4.13 Functional Genomics

Quantile-normalized gene expression was used to generate classifications linked to the limma-voom package in Galaxy Analysis (https://usegalaxy.org/), based on comparisons between high and low-SIVA1 expression groups. Differentially expressed genes (FC > 1.5; *p* < 0.05) obtained from Galaxy were used for gene ontology analysis with the ShinyGO 0.80 program (http://www.geneontology.org/). A heatmap was generated using ClustVis (https://biit.cs.ut.ee/clustvis/) to illustrate the 50 most differentially expressed genes between high- and low-SIVA1 expression samples.

### 4.14 Statistical Analyses

Statistical analyses were performed using GraphPad Prism 8. Comparisons between groups were conducted using Student’s *t*-test, one-way ANOVA, Kruskal–Wallis test, and Bonferroni post hoc test, as appropriate. Spearman correlation coefficients were calculated to assess the strength and direction of associations between *SIVA1* expression and genes of interest. Survival analyses were performed using the Kaplan–Meier method, Log-rank test, and Cox proportional hazards regression, comparing groups with low versus high *SIVA1* expression. Associations between *SIVA1* expression levels and breast cancer types or subtypes were evaluated using the chi-square test in IBM SPSS Statistics version 20 (IBM Corp.). For apoptosis, cell cycle, migration, invasion, and clonogenic assays, statistical analyses were performed using an unpaired, two-tailed Student’s *t* test to compare two independent groups (shControl vs shSIVA1; control vs treatment). Data are presented as mean ± SEM. Variance homogeneity was assessed using an F-test. The results were obtained from three independent experiments, and p-values <0.05 were considered statistically significant.

## Supplementary Material



## Data Availability

All data analyzed during this study are included in this published article and its Supporting Information files. The data supporting the findings of this study, including raw experimental data, spreadsheets used for data processing and statistical analyses, original gel/blot images (including biological and technical replicates used for quantification), and other source files generated during the course of the study, are available from the corresponding author upon reasonable request. These materials will be provided in a format that enables independent verification and reproduction of the reported results.

## Notes

*SIVA1* expression across molecular and histological subtypes of breast cancer in the TCGA cohort; association of *SIVA1* expression with breast cancer subtypes and survival outcomes; differential *SIVA1* expression between breast tumor and normal samples using TIMER 2.0 analysis; morphological analysis of breast cancer cells upon SIVA1 reduction; immunofluorescence analysis of SIVA1, actin cytoskeleton, and nuclei; histological classification and molecular subtypes of breast cancer cell lines; antibodies used for Western blotting; primers used for quantitative PCR (PDF)
