# Supplementary material for: SIVA1 Knockdown Drives Aggressive Phenotypes in Triple-Negative Breast Cancer Cells while Enhancing Paclitaxel Efficacy
Source: ACS Omega. 2026 Jun 20;11(26):39315–29. doi: 10.1021/acsomega.6c03785 (PMC13347369; doi:10.1021/acsomega.6c03785)
Supplement: Supplementary file 1 [file ao6c03785_si_001.pdf]

Supporting Information

SIVA1 knockdown drives aggressive phenotypes in triple-negative breast cancer cells while enhancing paclitaxel efficacy

Natália Sudan Parducci,[a,*] Bruna Oliveira de Almeida,[a] Anali Del Milagro Bernabe Garnique,[a] Maria Fernanda Lopes Carvalho,[a] Isabelle Diccini,[a] Leticia Veras Costa-Lotufo,[a] João Agostinho Machado-Neto,[a,*]

[a] Department of Pharmacology, Institute of Biomedical Sciences, University of São Paulo, São Paulo, CEP 05508-900, Brazil

***Corresponding authors:**
Natália Sudan Parducci, MSc
Email: nataliasudanparducci@gmail.com
João Agostinho Machado-Neto, PhD
Email: jamachadoneto@usp.br
Department of Pharmacology
Institute of Biomedical Sciences of the University of São Paulo
Av. Prof. Lineu Prestes, 1524, CEP 05508-900, São Paulo, SP, Brazil
Phone: 55-11-3091-7467; Fax: 55-11-3091-7322

Table of contents

**Figure S1**	**S-2**
**Figure S2**	**S-3**
**Table S1**	**S-4**
**Table S2**	**S-5**
**Table S3**	**S-6**

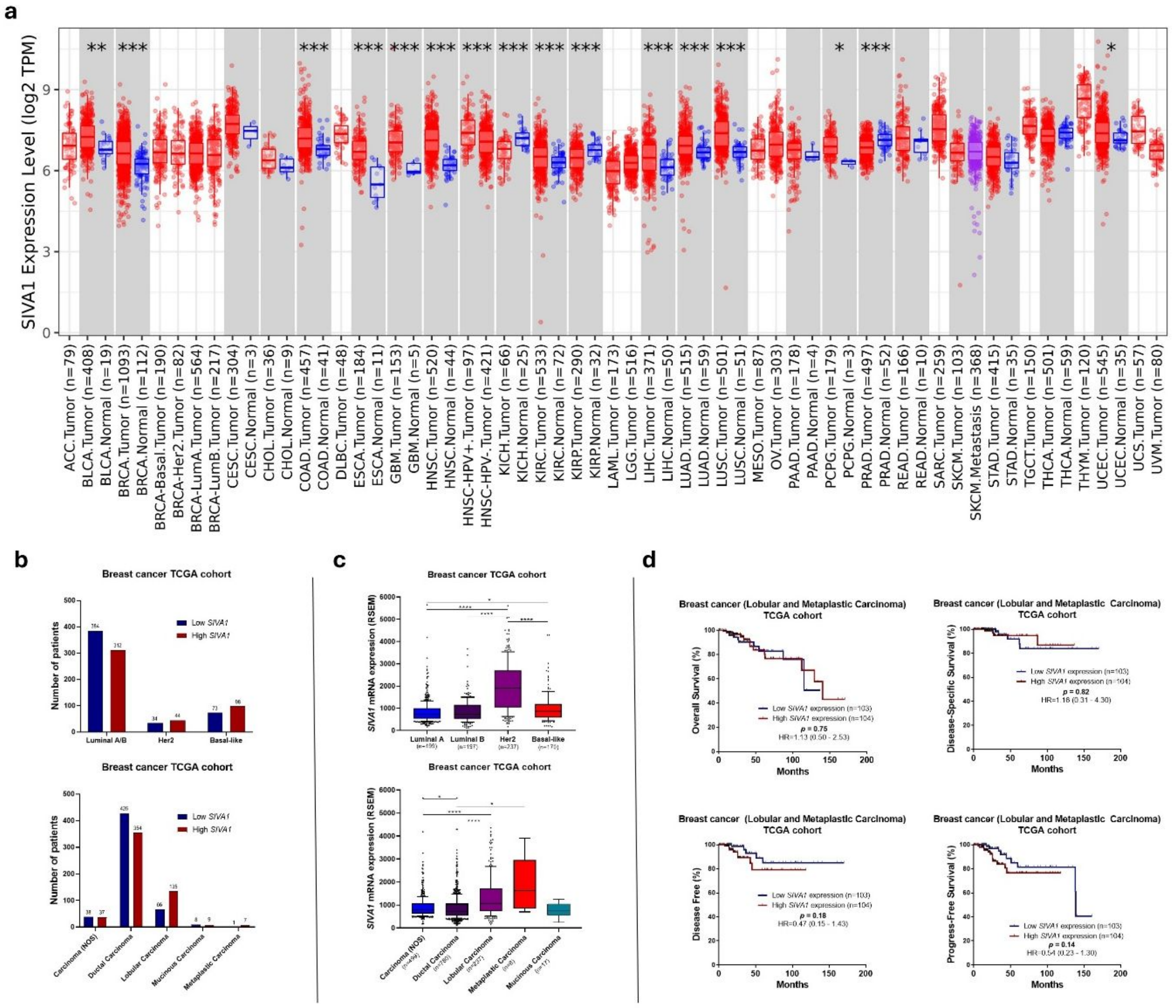

Supplementary Figure 1. *SIVA1* expression is increased in lobular and metaplastic breast cancer samples, and in subtypes Her2 and Basal-like. a Bar graphs showing the correlation between *SIVA1* expression and the frequency of molecular and histological subtypes of breast cancer in the TCGA cohort (*n*=1083) were generated using GraphPad Prism 8. Patients with low (blue) and high (red) *SIVA1* expression are highlighted. **b** The correlation between gene expression and molecular or histological subtypes was statistically assessed by the Kruskal–Wallis test. Increased *SIVA1* expression was associated with Her2, lobular carcinoma, and metaplastic subtypes. **c** Samples from the histological groups with the highest *SIVA1* expression were isolated, and their data were used to generate new survival plots. No statistically significant *p*-values were found for overall survival (*p*=0.75; HR=1.13), disease-specific survival (*p*=0.82; HR=1.16), disease-free survival (*p*=0.18; HR=0.47), or progression-free survival (*p*=0.14; HR=0.54). **d** Samples from BRCA.Tumor (*n*=1093) and BRCA.Normal (*n*=112) were analyzed using the TIMER 2.0 platform (http://timer.cistrome.org/). The gray columns indicate matched tumor and normal samples from 23 different cancer types, with tumor samples shown in red and normal samples in blue. Statistically significant differences in *SIVA1* expression were found in the breast cancer cohort (*p*<0.001). **p*<0.05, ***p*<0.01, ****p*<0.001. ANOVA and Bonferroni post hoc test. FDR threshold=0.05. BRCA, Breast Cancer. TCGA, The Cancer Genome Atlas.

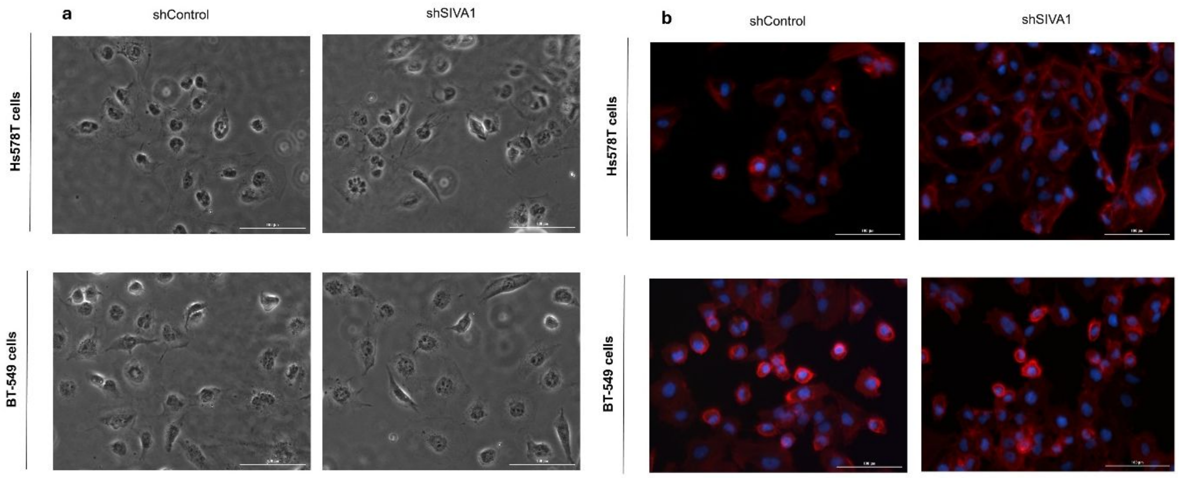

Supplementary Figure 2. Reduction in Siva1 levels does not alter the morphology of breast cancer cells. a Cell lines were seeded onto glass coverslips (Knittel glass), maintained in culture for 24 hours, and fixed with 4% paraformaldehyde solution. Three phase-contrast images per cell line were acquired using the LionHeart imaging system. **b** Fixed models were then permeabilized and stained with anti-Siva (1:100) and anti-rabbit Alexa Fluor 488 (1:600). Actin filaments were labeled with phalloidin conjugated to Alexa Fluor 647 (1:400). In the figure, Siva1 is shown in green, actin in red, and nuclei in blue (DAPI staining), with MERGE representing the overlay of all three signals. Scale bar=100 µm.

Table S1. Histological type and molecular subtype of breast cancer cell lines.

Cell line	Histological classification	Molecular subtype
MCF-10A	Non-tumoral	Immortalized mammary epithelial
MCF-12A	Non-tumoral	Immortalized mammary epithelial
MCF-7	Invasive ductal carcinoma	Luminal A
T-47D	Invasive ductal carcinoma	Luminal A
Hs578T	Invasive ductal carcinoma	Triple-negative
HCC70	Invasive ductal carcinoma	Triple-negative
HCC1937	Invasive ductal carcinoma	Triple-negative
BT-549	Invasive ductal carcinoma	Triple-negative
MDA-MB-231	Invasive ductal carcinoma	Triple-negative

*Data available from the American Type Culture Collection (ATCC).

Table S2. List of antibodies for Western blotting.

Supplier	Target	Species	Catalog Number
Cell Signaling Technology (MA, USA)	***Cytoskeleton***		
	Stathmin 1	Rabbit	13655
	p-Stathmin 1^{S16}	Rabbit	3353
Cell Signaling Technology (MA, USA)	***p53-mediated pathways***		
	p53	Rabbit	2527
	p-p53	Rabbit	82530
	p21	Rabbit	2947
	p73	Rabbit	14620
	Ubiquitin	Rabbit	20326
ThermoFisher Scientific (MA, USA)	***Siva1***		
	Siva1	Rabbit	5100737
Cell Signaling Technology (MA, USA)	***Cell death***		
	PARP1	Rabbit	9524
	$H2AX^{S139}$	Rabbit	9718
Cell Signaling Technology (MA, USA)	***Endogenous control***		
	α-tubulin	Rabbit	2144

Table S3. List of primers for qPCR.

Primer	Sequence (5´-3´)
ACTB	**FW:** CAT CCG TAA AGA CCT CTA TGC CAA C<br>**RV:** ATG GAG CCA CCG ATC CAC A
HPRT1	**FW:** GAA CGT CTT GCT CGA GAT GTG A<br>**RV:** TCC AGC AGG TCA GCA AAG AAT
SIVA1	**FW:** TCT TCG AGA AGA CCA AGC G<br>**RV:** TGC CCA AGC CTC CTG ATC
SIVA2	**FW:** CAG GAG GTC TTC GAC CCA<br>**RV:** AGT CCA CGA GGC CAC ACA
